# A New Illuminating Method by the Use of Zirconite

**Published:** 1892-06

**Authors:** W. R. Patton

**Affiliations:** Cologne, Germany


### A NEW ILLUMINATING METHOD BY THE USE OF ZIRCONITE.

BY DR. W. R. PATTON, COLOGNE, GERMANY.

GENTLEMEN,—I bring to your notice this, for us, new illuminating method, discovered or more properly worked out by Dr. Koch, Professor at the Bonn University, aided technically by the mechanician, Mr. Max Wolz, of the same place. I feel certain that I am introducing a means of great value to our particular specialty, for the various necessities which call for brilliant lighting effect. The inventive minds of our profession will rapidly develop its uses, and, by their facility of adapting ways to means, will undoubtedly lead to unforeseen advantages for its use in operative as well as for prosthetic dentistry.

My attention was attracted to this lighting method by a patient, a rising young medical practitioner, who had viewed and taken some interest in the ordinary reflecting petroleum lamp, the electric mouth-lamp made for our particular use; and I have consequently brought such an apparatus in order to demonstrate before you its powers, and in the hope of awakening your special interest to its peculiar worth and value. Everything necessary to explanation of this apparatus is from the papers of the discoverer, Dr. Koch, in the *Polytechnische Journal*, as well as from the *Pharmakologischen Institut* of Bonn. I feel assured of his satisfaction in my attempt to introduce to your notice the result of his research, which can be of such practical advantage to our specialty, and evade any future charge of plagiarism by acknowledging the fact beforehand. A few remarks relative to the production of light in general may not be taken amiss in order to verify the basis of this improvement and the causes which led to its development.

Since the introduction of the electrical arc light in illuminating technics, every means has been resorted to, and the powers of the inventor taxed to the utmost in the endeavor to heighten the light-effect of oils and gases, in order to at least approach the intensity of the electric. Great light-intensity, however, can only be arrived at through the corresponding high temperature of the light radiating body.

Solid bodies in darkness, at 400°, give but a weak light; a so-called dark-gray heat, at 600°, reaches a red heat, at 900° to 1000°, white heat,—whereas, gases even at 1500° to 2000° give no light,—at least not under ordinary circumstances. The flame temperature of the latter is consequently by introduction of air-combustion and by the heating of the burning gases, considerably increased. The flame itself thus becomes shorter, clearer, and hotter, because the burning process takes place more rapidly and less cold air comes in contact with the burning gases.

The glowing solid carbon in the gas or oil flame radiates from the hotter flame more light, and as in a smaller flame it is contracted into a smaller space the intensity of the light increases immensely.

The various regenerative lamps in use have (in this direction) reached all that is nigh posssible, but the light is, notwithstanding, still so yellow in its character that it cannot yet compete with the electric, which comparatively is the nearest to daylight in quality we have. From the preceding, one sees that for the production of intense white light, the hottest flame to get in connection with a solid glowing body, which is permanent in this temperature, must give theoretically the most perfect gas-light.

The highest possible temperature on this earth, to be reached by chemical processes, is that of a carbonic oxide flame burning in pure oxygen, and very near this point comes, under similar circumstances, the flames of hydrogen or coal-gas.

In past times such flames were much used for the production of the calcium light (Drummond's light) for light-house signalling and building purposes, and in the great American Civil War came into play for illuminating the field of action at the siege of various forts, and even now it is occasionally brought into requisition. Its use was encumbered with great drawbacks; the production of oxygen was costly and troublesome; the burners not appropriate, using too much gas; and in damp weather the lighting-body (pieces of cylindrical unslaked lime) fell to pieces, and on account of other disadvantages too numerous to mention, one particularly being the manufacture of the necessary oxygen very troublesome and expensive, its use was discontinued. Of later years, however, by the erection of factories for the production of oxygen, its use, particularly in England, has again become more general. Still, as every lime-light, in consequence of the rapid wearing away of the lighting-body (lime), requires the permanent attention and regulating of an individual, its use is more restricted to illuminating effect for short periods of time, as on the stages of theatres and for projecting lights.

The lime-light is particularly advantageous, and has often been made use of, in medical practice, for illuminating interior portions of the body, its high intensity and white light, by means of suitable reflectors, making it possible to define the finest differences of color of the mucous membranes, and so be of appreciable value in the diagnosis of disease in its various stages, which is impossible with the yellow oil or gas-light. As Dr. Koch, through some years of practical use for medical purposes, was constantly annoyed by the defects of the calcium-light, he was induced to experiment with *zirconearth* or *zirconite*, which finally led to the apparatus which will presently be exhibited and produced. *Zirconite* was discovered by Klaproth at the end of the last century, but Berzelius was the first to draw attention to the high power it possesses for emission of light. It is absolutely impossible to melt this *zirconite* by the highest possible chemical production of heat known up to date. Its power consists in accordance with its chemical purity, and the glow-body is manufactured by great pressure. Much experimenting has taken place in connection with the preparation of the zirconite body, but to Linnemann is accorded the credit of discovering that more success could be attained in manufacturing a burner for the coal-gas and oxygen flame, so that with the least possible gas and the least pressure the highest heat and light-effect could be secured. He demonstrated that the chemical union of both gases should take place one-half to one centimetre in front of the burner. In other words, the flame should burn in front of the burner and not at the burner. That this should constantly take place, the efflux or emanation of the mixed gases must be greater than the velocity of their explosion or the speed of their translation.

By a properly-constructed burner, the pressure of the oxygen must be fifteen times greater than that of the coal-gas, then there will be found, about one centimetre in front of the mouth of the burner, a light-blue, ball-shaped spot,—in reality the actual flame,—which possesses the highest temperature or heat, and the metal of the burner gets hardly warm, much less burnt on the point. If the oxygen pressure is less, the flame strikes backward,—that is, commences funnel-shaped at the oxygen emission-points, is less hot, and heats the burner in a short time very strongly. If the flame, as stated, be properly formed, then the entire induced caloric is concentrated at a small point some distance from the metal, and thus can, without any loss of power, work with full effect on the glow-body.

The Linnemann burner, however, notwithstanding its valuable qualities, was and is not adapted to medical purposes on account of its size and complexity, as well as its high cost. Credit is due to Dr. Koch and the mechanician, Mr. Wolz,—to the first through his preparation of the zirconite body, as well as the introduction of the glass tubes, to which I will later refer, and to the latter for the manufacture of a suitable burner, and to both for having arrived by combined labor in the production of a lamp or light of intense white heat, which, being of small volume, and being capable of concentration at one point, is of appreciable value to the specialist and microscopist. I will not take up your time with too much technical explanatory details, but merely allude to the leading points, such as the zirconite, which is prepared by fritting the zirconearth with a slight mixture of other material, and then by use of pressure. It can be, by his system, formed into homogeneous bodies of any size or shape. The zirconite is also porous, to better withstand the sudden and quick change of temperature, and yet sufficiently hard to be easily manipulated.

The Max Wolz burner is cheap and simple, possessing all the advantages of the Linnemann. It is so constructed that even when the pressure of the oxygen gas may somewhat vary the flame it is but slightly affected. The changeable friction of the gases, the peculiarly-constructed outlet, producing self-regulation, renders the burner practically useful. The dimensions are so chosen that with the smallest quantity of gas a maximum of light can be attained. It would not be economical to manufacture larger burners, for with a thicker central oxygen jet the mixture of the gases would be less perfect and unburned, and would very easily be lost on the hot glow-body. Such a defective burning of the flame can be recognized by looking at the illuminating point of the glow-body through a dark glass,—the spot where unburned oxygen reaches being dark or blackish.

I will now allude to another and one of the most valuable features of this illuminator,—viz., the glass-tube projectors, or better, the reflectors of the light, which are a revelation in their peculiarity and quite astounding as to the results of their capacity. A few remarks tending to explain the principle of their action will not be out of place. Their construction is based on the *total reflection*, which rays of light suffer under certain conditions, at the dividing surfaces between glass and air, when their tendency is from the glass towards the atmosphere. Those rays falling obliquely will, according to Lothe's laws of refraction, be diverted,—turned away. Now, as the diversion in the transmission from glass to atmosphere—bodies of great difference of density—is quite considerable, the oblique rays as a consequence will be reflected to the interior of the glass block again. This takes place by glass and air when the emission-angle is about $40\frac{3}{4}°$ and less. By recognition of these physical laws of total reflection, glass bodies can be produced, in which light, when once received, can be increased by continuous total reflection, until it reaches a surface at an angle of more than 42° from the receptive point, where it then issues. When light falls into or is conducted to a glass rod at a rectangular surface parallel to its length (or even slightly oblique), it cannot leave the rod, and must, when not partly absorbed by the glass, radiate as strong from the other end. The same takes place when the glass rod is so bent that the limits of total reflection are not overstepped.

Glass rods can be so prepared to evade light emanating from side-surfaces, so that the light can be led through perfectly pure glass with perfect side-surfaces with inconsiderable loss of power. The glass rod can be bent to a total inverted curve. As one end of a glass rod can be easily brought into the neighborhood of a strong light, and the other end close to an object, which will or cannot suffer a nearer approach to the light on account of its heat, you can perceive the advantages of this peculiarity of the lamp.

The glass rods must be specially prepared and chosen for the use of this reflection, as the ordinary ones in trade, when transversely transparent, are, in their parallel length, full of defects, and mostly permit only red or blue light to pass through, absorbing every other.

To the mechanician, Mr. Max Wolz, of Bonn, the manufacturer of this lamp, much praise is due for his trials and work in this direction until satisfactory results were reached.

